# Health communication, infodemics and disinformation about dementia related contents during COVID pandemic in Arabic countries: A bibliometric analysis

**DOI:** 10.1002/alz.086854

**Published:** 2025-01-09

**Authors:** Mohamed Taiebine, Abdelghafour Marfak, Chakib Nejjari

**Affiliations:** ^1^ Euro‐Mediterranean University of Fez (UEMF), Fez, Fez Morocco

## Abstract

**Background:**

The spread of fake news may lead to a disparate wave of digital health‐seeking behavior, cyberchondria, anxiety, indecision, and other psychosocial dysfunctions, including collapse in social capital and stigmatization. In this study, we utilized a bibliometric analysis to discern the primary trends associated with health communication and health‐seeking behavior regarding dementia‐related contents in countries within the Middle East and North African (MENA) region.

**Method:**

A literature review was conducted in November 2023. We examined articles published in English from 2020 to 2023 in PubMed, Web of Science, and Scopus databases that addressed health communication, infodemics, and disinformation during the COVID pandemic in Arabic countries. In total, 38 articles were identified. Four studies were selected based on the inclusion criteria. Bibliometric and thematic analysis have been performed by word frequency, geographical position, target population and language (given the diglossic status of Arabic‐speaking countries). The factors that contributed to the successful implementation of the socio‐behavioral and health belief models were described.

**Result:**

There is a dearth of research exploring content related to dementia or Alzheimer’s disease on social media in Arabic‐speaking countries. Several internal and external factors may influence health‐seeking behavior during the pandemic in Arabic‐speaking countries, including low levels of education and literacy, vulnerability to cognitive bias, lack of oversight from authorities, information overload, and an impulsive need to seek immediate responses without engaging in fact‐checking through social media.

**Conclusion:**

It is essential to implement a holistic framework to comprehend the behavior‐seeking attitudes of people with dementia based on socio‐cognitive and sociocultural beliefs during pandemics in order to protect vulnerable persons and provide the community with trustworthy and reliable information.

